# Supplementary material for: MetaRibo-Seq measures translation in microbiomes
Source: Nat Commun. 2020 Jun 29;11:3268. doi: 10.1038/s41467-020-17081-z (PMC7324362; doi:10.1038/s41467-020-17081-z)
Supplement: Supplementary file 10 — Supplementary Data 7 [file 41467_2020_17081_MOESM10_ESM.zip › File2/Confidence_High_Taxonomy/279457_out.krona.html]

Javascript must be enabled to view this page.

members
magnitude
magnitudeUnassigned
count
unassigned
taxon
rank

279457\_out

2

superkingdom
2
2

phylum
1239
2

186801
class
2

order
186802
2

2
family
186806

genus
1730
2


SRS016629\_contig\_number\_contig-100\_5689.33558SRS148784\_contig\_number\_28780
2
2293105
species
